# Pitfalls in Early Bioprocess Development Using Shake Flask Cultivations

**DOI:** 10.1002/elsc.70001

**Published:** 2025-01-28

**Authors:** Gesa Brauneck, Dominik Engel, Luca Antonia Grebe, Maximilian Hoffmann, Philipp Georg Lichtenberg, Anne Neuß, Marcel Mann, Jorgen Barsett Magnus

**Affiliations:** ^1^ AVT – Biochemical Engineering RWTH Aachen University Aachen Germany

**Keywords:** biochemical engineering, bioprocess development, cultivation system, online monitoring, shake flask

## Abstract

For about 100 years, the shake flask has been established for biotechnological cultivations as one of the most important cultivation systems in early process development. Its appeal lies in its simple handling and highly versatile application for a wide range of cell types—from bacteria to mammalian cells. In recent decades, extensive research has been conducted on the shake flask, to not perform processes blindly but to gain a deeper understanding of the various process parameters, phenomena, and their impact on the process. Although the characterization of the shake flask is now well‐established in literature, many publications show that this knowledge is often inadequately applied. Therefore, this review provides an overview of the current state of knowledge on various topics related to the shake flask. We first present the key process parameters and their influence on different physical phenomena, such as power input, the largely unknown in‐phase/out‐of‐phase phenomenon, as well as temperature and mass transfer. Then, the most common online monitoring systems that have been established for shake flasks are discussed. Finally, various pitfalls that often arise from inadequate knowledge of handling shake flask cultivations are discussed and guidance on how to avoid them is provided.

**Abbreviations**
CTR: carbon dioxide transfer rate
DOT: dissolved oxygen tension
OTR: oxygen transfer rate
OTR_max_
: maximum oxygen transfer rate
RAMOS: respiration activity monitoring system
RQ: respiratory quotient

## 1 Introduction

At the beginning of the 20th century, the method of submerged cultivation was established for microbial cultivations [1, 2]. Before that, liquid media were only used for chemical reactions as biological cultivations were conducted on solid growth media in Petri dishes [3]. The first reports about shaken cultivations in flasks were published by Kluyver and Perquin in 1933 [4]. In their work, they developed a submersed cultivation method for mold using shake flasks [1, 4]. One of the first production processes in shake flask with industrial relevance used *Penicillium chrysogenum* to produce the antibiotic penicillin [5].

**FIGURE 1:**
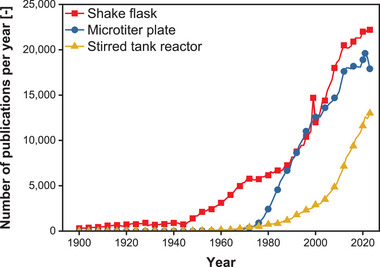
Development of number of publications per year on commonly used systems for biotechnological cultivations between 1900 and 2023. Google Scholar searched for “shake flask”, “microtiter plate”, and “stirred tank reactor”. The search was made on Jul 16, 2024, using https://scholar.google.com/. For clarity, only every fourth data point is plotted as a symbol. Lines are drawn through all data points.

Shake flasks were not only an important utensil for cultivations on a laboratory scale in the past but are still a commonly used tool today. As Figure 1 shows, the number of publications in which the use of shake flasks is mentioned has been steadily increasing since the 1940s—shortly after the first submerged cultivation in flasks and its first large‐scale process in the industry—until today. With more than 20,000 publications per year, the highest number was reached in 2023. The data of two other widely used cultivation systems in laboratory‐scale research—the microtiter plate and the stirred tank reactor—is shown for comparison. It is evident that the use of these two cultivation systems was only adopted in research at a later stage, as publications only started to be published after 1970. The shake flask has not been displaced; all three cultivation systems are still in use today since the number of publications is continuously increasing. However, it must be considered that the publication search did not differentiate between the various applications of the three cultivation systems. They can be used not only for biotechnological processes but also for abiotic applications, such as enzymatic assays or processes in the chemical industry. Nonetheless, this predicament occurs for all three cultivation systems, so the basic statement regarding the increasing number of publications can still be made.

Shake flasks are primarily used in early process development and as a screening platform. For example, the composition of cultivation media or different process parameters, such as the pH value or temperature, are often screened in shake flask cultivations [6, 7]. Reasons for the frequent and versatile application are the high throughput of experiments compared to stirred tank reactors and the simple handling of cultivations in shake flasks [8]. Compared to other small‐scale cultivation platforms, such as microtiter plates, shake flasks offer the advantage of a larger liquid filling volume, allowing for more comprehensive analysis [6, 9]. As demonstrated in the following chapters, shake flasks are also much better characterized in terms of physical effects and phenomena, for example, power input, compared to microtiter plates, which is crucial for successful scale‐up. Moreover, shake flask cultivations have a low material requirement and can be used flexibly in many different applications—bacteria, yeast, fungi, plant cells, mammalian cells, and others [6, 10, 11, 12, 13, 14, 15, 16, 17]. All these factors make shake flask cultivations a cost‐efficient platform for process development in terms of working time and material use.

Nowadays, shake flasks are one of the most, if not the most used utensils, for primary screening and early process development. While 25 years ago, many phenomena and parameters in shake flask cultivations were still unknown [14], the processes are very well characterized today. Unfortunately, as numerous publications indicate, this knowledge is frequently disregarded in most shake flask cultivations, which we seek to counteract with this review. First, we present the most important characteristics and phenomena in shake flask cultivations that have been described over the last decades. This is followed by an overview of various online measurement techniques. They simplify process characterization and offer the possibility to online monitor a variety of measurement variables. A subsequent chapter provides advice on what lessons can be learned from these theoretical considerations and which pitfalls should be avoided in future experiments.

## 2 Characteristics of Shake Flasks

**FIGURE 2:**
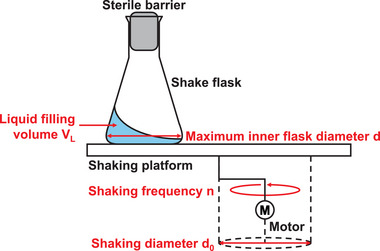
Setup of a shake flask cultivation. Process parameters—liquid filling volume, maximum inner flask diameter, shaking frequency, and shaking diameter—are written in red.

To ensure a consistent understanding of the physical effects and phenomena presented in the following chapters, the design of a shake flask and its key process parameters are briefly outlined below. For illustration, the process parameters are shown in Figure 2.

### 2.1 Design of Shake Flasks

Shake flasks are Erlenmeyer flasks, developed in 1860 [18], that are used for biotechnological processes. The flask is characterized by the flat round base, making it stable for shaken cultivation experiments, the conical shape, and the tapered shape towards the neck of the cylinder. Additionally, the shape can be varied by inserting baffles leading to turbulent flow behavior. The shape and number of baffles are versatile and depend on the manufacturer and the application [19]. In this review, only unbaffled shake flasks are considered. The reasons for this are the otherwise resulting complexity of the individual topics and the problems associated with baffled shake flasks which are discussed in Chapter 5.1.2. Shake flasks are typically made out of glass, more precisely borosilicate glass [20]. There are also shake flasks that are manufactured using plastic materials, for example, polycarbonate or polypropylene [21, 22]. Glass is more common due to its stability against heat and most solvents and inert properties during chemical and biological reactions [20]. The volume of shake flasks varies between 25 and 5,000 mL [14]. A lower volume is not suitable due to high relative volume losses connected to condensation effects in smaller flasks. Flasks with a volume of more than 5,000 mL would not be suitable in typical laboratory shakers due to their size. The neck of a shake flask is usually sealed with a sterile barrier to protect the medium inside the shake flask from contamination and prevent condensation of the liquid. Different kinds of sterile barriers are used, such as plugs made of cotton or foam, aluminum foil or parafilm, silicone caps, clip‐on aluminum caps, and screw caps [17, 23]. In addition, to the separation of the inside of the flask from the environment, another requirement for the sterile barrier is sufficient gas permeability to ensure the supply of gas to the culture [21, 23].

### 2.2 Process Parameters of Shake Flask Cultivations

Shake flasks rely on mechanical agitation of the flasks to ensure proper mixing, oxygen transfer, and nutrient distribution, which are crucial for optimal microbial growth and metabolite conversion. During a process using shake flasks, several parameters can be varied which have an impact on different phenomena and the performance of the process. The most common process parameter that is varied for shake flask cultivations is the shaking frequency (n). It describes the speed at which the shake flask is shaken. Depending on the application, it is set up to 400 rpm [24, 25, 26]. There are two different modes of shaking, orbital and linear, whereby nowadays almost only orbital shaking is used [14, 17]. Moreover, the shaking diameter (d_0_)—the orbit diameter of the circular path that the shaking platform follows when it moves in a shaking motion—can be varied. For shake flask cultivations, it is normally set to values up to a few centimeters. The size and therefore also the total volume of the flask is mostly characterized by its maximum inner flask diameter (d). It is the only process parameter that can be changed for the shake flask itself (if flasks are used according to standard geometry). Additionally, the liquid filling volume (V_L_) of the flask can be varied. According to a rule of thumb, 5 % to 10 % of the flask volume is typically used as an optimal liquid filling volume for microbial cultivations [27, 28]. Parameters to be varied beyond this, such as media composition and incubation time, will not be further discussed because they are not specific to a process in shake flasks but also apply to other cultivation systems.

## 3 Physical Effects and Phenomena of Shake Flask Cultivations

As this review focuses on cultivations in shake flasks, physical effects and phenomena that are important in this cultivation system are presented below. The focus lies on phenomena that either occur exclusively in shake flask cultivations or that differ greatly from other scales, such as the stirred tank reactor. Other effects that have a similar consequence regardless of the scale are not considered.

### 3.1 Power Input and Hydromechanical Stress

Power input is a crucial engineering parameter and important for comparing cultivation conditions in different vessels [29]. The volumetric power input (P/V) describes the power input per liquid volume into a cultivation vessel. For shake flasks, the average volumetric power input (P/V)_Ø_ can be calculated according to Equation (1) which also comprises the liquid density (ρ) [30, 31].

(1) $$\;{\left(\frac{P}{V}\right)}_{Ø}=\;{\mathrm{Ne}}^{\prime }\cdot \rho \cdot \frac{n^{3}\cdot d^{4}}{V_{L}^{2/3}}\;$$

The modified Newton number for shake flasks (Ne’) can be calculated using Equation (2) including the Reynolds number (Re), see Equation (3) [31]. To calculate the Reynolds number, also the dynamic viscosity (*η*) must be considered.

(2) Ne′=70·Re−1+25·Re−0.6+1.5·Re−0.2

(3) Re=ρ·n·d2η

As becomes obvious from Equation (1), the average volumetric power input in shake flasks is easily adjustable by varying key cultivation parameters like shaking frequency, vessel size as well as shaking diameter, and liquid filling volume. This can be used, for example, to synchronize cultivation conditions between different cultivation vessels, and, thus, for scale‐up and scale‐down approaches.

Another important parameter describing the liquid flow within a vessel is the energy dissipation rate. The average energy dissipation rate (*ε*_Ø_) describes the irreversible conversion of kinetic energy to heat and is calculated according to Equation (4) [32].

(4) $$\;\varepsilon_{\emptyset }={\left(\frac{P}{V}\right)}_{\emptyset }\;\cdot \frac{1}{\rho }$$

From stirred tank reactors, it is well known that the average energy dissipation rate must be distinguished from the maximum local energy dissipation rate (ε_max_). However, in shake flasks, the average energy dissipation rate is equal to the maximum local energy dissipation rate because it is evenly distributed and therefore has no local maxima. This applies to laminar flow regimes. The calculation of the energy dissipation rate in shake flasks was in detail studied by Peter et al. [33]. The most important, simplified equations are summarized in Equations (5) to (9) with the liquid height of the bulk liquid inside the flask (h) and the axial Froude number (Fr_a_) [8].

(5) $$\mathrm{Laminar}\;\mathrm{flow}\;\mathrm{regime}:\;\varepsilon_{\max}=\varepsilon_{\emptyset }$$

(6) $$\mathrm{Turbulent}\;\mathrm{flow}\;\mathrm{regime}:\varepsilon_{\max}=0.1\;\frac{{\left(\pi \cdot n\cdot d\right)}^{3}}{h}$$

(7) $$\mathrm{with}\;{\mathrm{Fr}}_{a}\;>\;0.4:\;h=1.11\cdot d_{0}^{0.18}\cdot d^{-0.11}\cdot \;n^{0.44}\cdot V_{L}^{0.34}$$

(8) $$\mathrm{with}\;{\mathrm{Fr}}_{a}\;\leq \;0.4:\;h=1.31\cdot d_{0}^{0.28}\cdot d^{0.02}\cdot n^{0.9}\cdot V_{L}^{0.35}$$

(9) $$F\;r_{a}=\frac{{\left(2\cdot \pi \cdot n\right)}^{2}\cdot d_{o}}{2\cdot g}\;$$

The flow regime was defined as laminar for Re < 60,000 and turbulent for Re > 60,000 [33].

The maximum local energy dissipation rate or the quotient *ε*_max_/*ε*_Ø_ is one parameter quantifying the phenomenon of hydromechanical stress. Hydromechanical stress encompasses various types of stresses and results from the movement or flow of fluids [34, 35]. There are different methods to determine the maximum local energy dissipation rate. Indirect measurements are shear‐sensitive systems like the maximum stable drop size [34, 36, 37, 38], flocculation systems [35, 39], or shear‐sensitive layer aggregates [40]. For shake flasks, fundamental basic research was done with the maximum stable drop size method [33]. Some of the most important findings include the following: The maximum local energy dissipation rate is not influenced by the liquid filling volume and is more than one order of magnitude lower than in stirred tank reactors.

### 3.2 In‐Phase/Out‐of‐Phase Phenomenon

In‐phase behavior in shake flasks is defined as the orbital motion of the liquid within the flask being synchronized with the flask movement. Consequently, it is a phenomenon that can only occur in shaken and not in stirred systems. It leads to uniform mixing and gas transfer, resulting in consistent optimal conditions for microbial cultivations. Cultures will be in‐phase if the power input exceeds certain thresholds [30, 31]. If the power input is not sufficient, the bulk liquid might become out‐of‐phase. This out‐of‐phase phenomenon was introduced by Büchs et al. [41]. It was described as the oscillatory motion of the bulk liquid within the flask not being synchronized with the movement of the flask, resulting in undefined, heterogeneous conditions as well as limited oxygen transfer and mixing [42].

The suitable adjustment of key cultivation parameters, such as shaking frequency, flask size, liquid filling volume, and the type of shaking motion, is crucial to prevent the out‐of‐phase phenomenon. The Phase number (Ph) is a critical parameter to quantify the possibility of an out‐of‐phase phenomenon occurring. The Phase number, often referred to as the phase angle or phase shift, describes the extent to which one waveform or oscillation is ahead or behind another in its cycle. It can be calculated according to Equation (10). This equation applies to a wide range of operating conditions and is based on the correlation for the power input [31].

(10) $$\mathrm{Ph}\;=\frac{d_{0}}{d}\;\cdot \left(1+3\cdot \mathrm{lo}g_{10}\left[\frac{\rho \cdot n\cdot d^{2}}{\eta }\cdot \frac{\pi }{2}\cdot {\left(1-\sqrt{1-\frac{\pi }{4}\cdot {\left(\frac{V_{L}^{1/3}}{d}\right)}^{2}}\right)}^{2}\right]\right)$$

It was determined that an out‐of‐phase phenomenon can be prevented with Ph > 1.26 and Fr_a_ > 0.4. If these values are exceeded, the conditions are determined to be in‐phase without the undesired effects attributed to an out‐of‐phase phenomenon [43, 44, 45, 46]. Advancements in computational fluid dynamics enable a detailed examination of liquid distribution in shake flasks with waterlike to moderate viscosities. This allows for precise determinations of in‐phase/out‐of‐phase phenomenon [47]. Additionally, the energy dissipation in the form of the volumetric power input can be calculated with high accuracy, which serves as a critical parameter for translating bioprocess performance across different scales, from shake flasks to industrial stirred tank reactors.

### 3.3 Heat Transfer

Temperature is an important parameter in cultivations, and it is influenced by the formation, transfer, and release of heat. The specific heat generation (Q̇) in the medium is caused on the one hand by the volumetric power input into the medium and on the other hand by the metabolic activity of the microorganisms (Q̇_met_), see Equation (11) [48].

(11) $$\;\dot{Q}=\left(\frac{P}{V}\right)+{\dot{Q}}_{\mathrm{met}}$$

Thermodynamically, metabolic heat generation corresponds to the change in Gibbs energy (ΔG) resulting from the various metabolic reactions (under the assumption of constant temperature and pressure as well as neglectable entropy (S) changes) [49, 50]. Reactions only take place spontaneously when the Gibbs energy decreases. However, the biosynthesis of complex molecules, such as proteins, nucleic acids, lipids, peptidoglycan, other products, and cell components, is mostly endergonic and requires energy supply (ΔG > 0). The necessary energy is provided by the metabolism of energy‐rich molecules, for example, glucose, whose catabolic reactions are so exergonic (ΔG << 0) that the total change in Gibbs energy is negative (ΔG < 0) [48, 49].

According to Equation (12), the change of the Gibbs energy depends on the change in enthalpy (H) and entropy of the system. Under aerobic growth, the entropy changes due to catabolic reactions and biosynthesis can be neglected, so that the heat generated by the metabolic activity corresponds to the enthalpy change, see Equation (13) [50].

(12) ΔG=ΔH−T·ΔS

(13) $$\;{\dot{Q}}_{\mathrm{met}}=\;\Delta H$$

The generated heat is released into the environment via the surface of the liquid and shake flask. According to Equation (14), the heat flow depends on the surface area (A), the respective heat transfer coefficient (k), and the difference in temperature (T) [48]. To keep the temperature of the medium constant during cultivation, the ambient temperature of the shake flask can be controlled using a temperature‐controlled hood.

(14) $$\;\dot{Q}=A\cdot k\cdot \Delta T$$

### 3.4 Mass Transfer

Oxygen is the most critical substrate for aerobic cultivations. Due to its low solubility in aqueous media, a constant supply of oxygen must be guaranteed to ensure non‐limiting conditions. Various transfer resistances must be overcome in shake flask cultivations to ensure an optimal oxygen transfer rate (OTR) [51].

First, the transfer resistance of the sterile plug must be overcome. The OTR through the plug (OTR_plug_) depends on the respective transfer coefficient (k_plug_), the oxygen partial pressures inside (p_O2,in_) and outside (p_O2,out_) of the shake flask, and the total pressure (p_abs_), see Equation (15) [52]. According to the model of Henzler and Schedel, shown in Equation (16), the transfer coefficient through the plug depends on the diffusion coefficient of the respective plug material (D_O2,e_), the cross‐sectional area of the flask neck (A_F_), the height of the sterile plug (h_plug_), and the molar gas volume (V_m_) [52, 53]. Alternatively, the transfer coefficient through the plug can be determined experimentally [23].

(15) $$\;\mathrm{OT}R_{\mathrm{plug}}=k_{\mathrm{plug}}\cdot \frac{p_{O_{2},\mathrm{out}}-p_{O_{2,\mathrm{in}}}}{V_{L}\;\cdot \;p_{\mathrm{abs}}}$$

(16) $$\;k_{\mathrm{plug}}=\frac{D_{O_{2},e}\cdot A_{F}}{h_{\mathrm{plug}}\cdot V_{m}}\;$$

The largest transfer resistance lies at the interface between the gas phase and the liquid phase. According to Equation (17), the OTR through the interface (OTR_G–L_) depends on the mass transfer coefficient (k_L_), the specific transfer area (a), and the difference in the molar concentration of oxygen between the interface (c^*^_O2,L_) and in the liquid (c_O2,L_) [8, 26]. At the interface between the two phases, an equilibrium is reached in which the oxygen concentration in the liquid corresponds to the oxygen partial pressure of the gas. Thus, it can be described by the difference in oxygen partial pressure at the interface (p^*^_O2,G_) and in the gas phase (p_O2,G_) [8, 26, 52, 54]. These differences can be converted to the mole fraction at the interface (y^*^_O2,G_) and in the gas phase (y_O2,G_) using the oxygen solubility (L_O2_), shown in Equation (18) [26].

(17) $$\;\mathrm{OT}R_{G-L}=k_{L}a\cdot \left(c_{O_{2},L}^{*}-c_{O_{2},L}\right)$$

(18) $$\begin{array}{cc} \mathrm{OT}R_{G-L} & =k_{L}a\cdot L_{O_{2}}\cdot \;\left(p_{O_{2},G}-p_{O_{2},G}^{*}\right)\\ & =k_{L}a\cdot p_{\mathrm{abs}}\cdot L_{O_{2}}\cdot \left(y_{O_{2},G}-y_{O_{2},G}^{*}\right) \end{array}$$

When the oxygen concentration in the liquid drops to zero, the OTR becomes maximum (OTR_max_) and it is calculated using Equation (19) [25, 55]. The OTR_max_ always describes the maximum transfer rate depending on the physical system and not on the biological system. To prevent the cultivation from oxygen limitations, the maximum oxygen uptake rate of the microorganisms must not exceed the OTR_max_ [55].

(19) $$\;\mathrm{OT}R_{\max}=k_{L}a\cdot p_{\mathrm{abs}}\cdot L_{O_{2}}\cdot y_{O_{2},G}$$

The individual parameters can be adjusted to increase the OTR_max_. However, a change in the total pressure and the oxygen mole fraction in the gas phase is only possible by applying overpressure or aeration with gas mixtures with increased oxygen concentrations. Both can be dangerous in practical work due to explosion risk and oxygen toxicity, respectively. The solubility and the mass transfer coefficient value strongly depend on the respective medium and its salt concentrations [25]. The specific mass transfer area, on the other hand, can be influenced by the shaking conditions. These include the shaking frequency, the liquid filling volume, the shaking diameter, and the shake flask diameter. Seletzky et al. investigated the oxygen transfer by means of a sulfite system and described a correlation, according to which these parameters influence the volumetric mass transfer coefficient (k_L_a) as shown in Equation (20) [55].

(20) $$k_{L}a=6.67\cdot 10^{-6}\cdot n^{1.16}\cdot V_{L}^{-0.83}\cdot d_{0}^{0.38}\cdot d^{1.92}$$

Using Equation (20) and the results from 343 biotic and abiotic measurement experiments on OTR_max_, Meier et al. adapted the equation for calculating the OTR_max_. The media composition in the form of the osmolality (osmol) of the medium is used as a parameter in the calculation [25]. To take its influence on the shaking frequency into account, the exponent of the shaking frequency was also adjusted accordingly, see Equation (21).

(21) $$\begin{array}{cc} \mathrm{OT}R_{\max} & =3.72\cdot 10^{-7}\cdot \mathrm{Osmo}l^{0.05}\cdot n^{\left(1.18-\frac{\mathrm{Osmol}}{10.1}\right)}\\ & \;\cdot V_{L}^{-0.74}\cdot d_{0}^{0.33}\cdot d^{1.88}\cdot p_{\mathrm{abs}}\cdot y_{O_{2},G} \end{array}$$

The resistances during the following oxygen transfer through the liquid to and into the cells are negligible compared to the resistance through the interface. Only cell agglomerations can lead to increased resistance, which reduces oxygen transfer [51].

## 4 Online Monitoring Devices

To obtain better control and understanding of shake flask cultivations, various methods for online monitoring of important measurement variables have been developed in recent years [8, 14]. Such measurables can describe the liquid phases—pH value, dissolved oxygen tension (DOT), viscosity, and biomass—or the gas phase—OTR, carbon dioxide transfer rate (CTR), and respiratory quotient (RQ). The most frequently used technologies for online monitoring of these measurables are presented below.

### 4.1 Online Monitoring of the Liquid Phase

Multiple approaches have been explored to implement traditional probes for DOT and pH value monitoring, typically employed in stirred tank reactors, within shake flasks. However, they are unsuitable for routine application. The installation of the probe creates a baffle, which significantly changes the hydrodynamics inside of the shake flask, thereby increasing the OTR_max_ [56]. Although this seems advantageous at first, the results from the monitored shake flask are not transferable to a standard, unbaffled flask [57]. Also, shake flasks with commonly used sizes provide limited space, allowing for only one or two probes to be installed. Moreover, conventionally available probes are not designed to endure the shaking motion and will quickly be damaged. For the aforementioned reasons, less invasive and non‐invasive online monitoring systems for different cultivation parameters in shake flasks were developed.

#### 4.1.1 Online Monitoring of the pH Value

A crucial parameter that is typically measured in biotechnological processes is the pH value. In shake flasks, the pH value can be determined using fluorescent pH‐sensitive spots, which are attached to the inside of the flask and read out optically from the outside [58]. The spots cover a range of two to four pH value units and consist of two fluorescent dyes. One dye is pH value insensitive, whereas the other dye shows a pH value sensitive fluorescence behavior. Both are excited by light and exhibit luminescence in the near‐infrared region. If the pH indicator is deprotonated at a high pH value, its fluorescence will be quenched, resulting in the measurement of only the near‐infrared emission of the reference indicator. If the pH value decreases, the pH indicator dye will be protonated, resulting in the emission of near‐infrared luminescence. The excitation light is sinusoidally modulated, resulting in a phase‐shifted sinusoidally modulated emission in the near‐infrared spectrum depending on the pH value. The attached read‐out device measures this phase shift, which is then converted into the pH value. These spots are nowadays commercially available (pH Sensor spots, PyroScience, Aachen, Germany). Other devices commercially available devices that measure the pH value along with additional parameters are discussed in the following chapters about online monitoring of dissolved oxygen and biomass.

#### 4.1.2 Online Monitoring of the Dissolved Oxygen

To describe the amount of dissolved oxygen within the liquid phase, the DOT is typically used. It can be determined similarly to the pH value using fluorescent, oxygen‐sensitive spots. Hansen et al. investigated the applicability of these DOT spots in shake flasks [57]. They found that the use of DOT spots at fixed positions in the shake flask is not suitable in cultivations with low liquid filling volumes and high shaking frequencies. Due to the rotation of the shake flask, the spot is not in continuous contact with the bulk liquid over the whole course of one revolution but is only covered with a thin film of liquid for the majority of the time. Because of the slow reaction time of the DOT spots, a mixed signal is produced, which results in erroneous measurements [56, 57]. In contrast to DOT spots, pH value spots do not require continuous contact with the liquid, as contact with the liquid film is sufficient for reproducible measurements. An alternative option to the use of fluorescent oxygen‐sensitive nanoparticles can be applied, as presented by Borisov and Klimant [59]. The nanoparticles are added directly to the cultivation medium to ensure continuous contact with the liquid. Flitsch et al. and Ladner et al. demonstrated the applicability of commercially available oxygen‐sensitive nanoparticles (Oxnano, PyroScience, Aachen, Germany) in shake flask cultivations [56, 60]. The nanoparticles contain oxygen‐sensitive dyes and have a hydrophilic vinylpyrrolidone shell. The measurement principle relies on the oxygen‐depending quenching behavior of the fluorescent dyes which is read out by an optoelectronic sensor [59]. The optical sensor remains outside of the shake flask and alternately determines the DOT in the rotating liquid and the liquid film [56, 60]. For precise measurements, it is essential to take the angular position of the shaking platform into account. Based on the reasons outlined, the use of the added oxygen‐sensitive particles appears to be advantageous compared to the spots. Nowadays, various devices are commercially available that combine measurements of pH value, DOT, and—depending on the manufacturer—other measurement variables (Shake Flask Reader vario, PreSens, Regensburg, Germany; Firesting, PyroScience, Aachen, Germany; Multiparameter Sensor, Scientific Bioprocessing, Baesweiler, Germany). The Shake Flask Reader vario, in addition to measuring the DOT, also allows for pH value, OTR, and biomass measurements. Its key advantage is its versatility, enabling the monitoring of various vessel types, such as T‐flasks or cultivation tubes. However, a notable drawback is the need for pre‐calibrated sensor spots and specific flasks. The Firesting measures the pH value and oxygen in both liquid and gas phases, making it applicable to lots of different applications next to biotechnological cultivations. The most versatile device is the Multiparameter Sensor, as it measures biomass, fluorescence, and DOT.

#### 4.1.3 Online Monitoring of the Viscosity

Ladner et al. also used the rotation of the bulk liquid to measure the viscosity of the medium in shake flask experiments [60]. The position of the bulk liquid relative to the centrifugal force depends on the viscosity of the liquid. With increasing viscosity, there is a shift of the bulk liquid relative to the centrifugal force in the direction of shaking. This shift was first used by Sieben et al. to determine the viscosity [61]. To determine the position signal for the centrifugal force, a Hall effect sensor was used, which was combined with light transmission measurements in the near‐infrared range to track the position of the leading edge of the bulk liquid. Thereby, it is possible to monitor the viscosity of the liquid in shake flasks contact‐free and online, creating a viscosity monitoring system. Ladner et al. used the same principle to determine viscosity in shake flask experiments. However, the position of the leading edge of the rotating bulk liquid was not determined by light transmission measurements but by utilizing the fluorescence signal intensity of oxygen‐sensitive nanoparticles. In addition, tracking of the position of the bulk liquid facilitates triggered DOT measurements of the oxygen‐sensitive nanoparticles leading to better measurement accuracy [60].

#### 4.1.4 Online Monitoring of the Biomass

As a measure of biomass, scattered light can be determined online. This has proven to work reliably for microtiter plates and the results are comparable to offline optical density measurements [62]. Some studies, extend this non‐invasive online monitoring of the biomass using scattered light, in particular backscattering, to the shake flask scale. Similar to DOT measurements with nanoparticles, the scattered light measurement in the shake flask is strongly influenced by the position of the rotating bulk liquid. Ebert et al. investigated the influence of the liquid filling volume, shaking frequency, and measurement duration in different shake flasks on the scattered light signal [63]. Nowadays, online measurement techniques for biomass monitoring in shake flasks are commercially available (Cell Growth Quantifier, Scientific Bioprocessing, Baesweiler, Germany; Shake Flask Reader vario, PreSens, Regensburg, Germany; Multiparameter Sensor, Scientific Bioprocessing, Baesweiler, Germany). The advantages and disadvantages of the Shake Flask Reader vario and the Multiparameter Sensor have already been outlined in Chapter 4.1.2. The Cell Growth Quantifier eliminates the need for sensor spots and is compatible with a wide range of existing flasks in various sizes and geometries. Although it is limited to cell growth measurement, it can be integrated with other measurement systems. In the most recently published device ShakeVisc, already available pH sensor spots and oxygen‐sensitive nanoparticles for DOT measurement are combined with a scattered light sensor and viscosity monitoring in a single system for shake flasks which enables an unprecedented number of online signals [64]. However, this measuring device is not yet commercially available.

### 4.2 Online Monitoring of the Gas Phase

The understanding and control of gas transfer is a central part of biotechnological process management. Particularly the oxygen transfer is important, as it plays a crucial role in cellular metabolism and productivity. The oxygen transfer capacity dictates the oxygen availability in the cultivation, which is vital for aerobic organisms. Inadequate oxygen transfer can lead to suboptimal cultivation conditions, affecting both cell growth and product formation [65]. Commercial exhaust gas analyzers for fermenter‐scale cultivations calculate the OTR based on the oxygen concentration difference between the inlet and outlet gas stream [66]. They therefore require a high measurement accuracy and precision and are prone to drifting. For parallelized shake flask scale cultivations, a different measurement principle can be employed. The respiration activity monitoring system (RAMOS) is an intermittent online measuring system enabling the measurement of the OTR in up to eight shake flasks. During the measuring phase, the OTR is calculated from the decrease of the oxygen partial pressure in the headspace of the shake flask [67]. RAMOS is a versatile system and can be altered to suit various applications spanning aerobic and anaerobic cultivations, microorganisms, plant cells, and mammalian cells. An overview of application examples and relevant literature is summarized in Table 1. Devices employing this measurement principle are nowadays commercially available (RAMOS, Hitec Zang GmbH, Herzogenrath, Germany; Kuhner TOM, Kuhner Shaker GmbH, Herzogenrath, Germany). Both commercially available systems are comparable in their basic measuring principle and differ more in their instrumental measuring setup. The advantage of the Kuhner TOM system is its modular and space‐saving design, which allows it to be retrofitted in many shakers. Handling is also easier, as only a cap needs to be connected to the flask.

**TABLE 1:** Versatile use of the RAMOS for measurement of the gas phase in shake flask cultivations.

Monitored organism	Application example	Literature
Microorganisms, for example, bacteria, yeasts, and fungi	Medium optimization, optimal time of harvest	[6, 67, 68, 69]
Mammalian cells	Time‐resolved determination of key culture parameters in Chinese hamster ovary cell cultivations	[70, 71, 72]
Plant cell suspensions	Identification of priming compounds, medium optimization for protein production	[73, 74]
Whole plants	Indication of plant viability, day/night rhythm	[75]

Monitored gas	Application example	Literature
Oxygen	Identification of gas transfer limitation, correlation to substrate consumption	[9, 67, 69]
Carbon dioxide	Anaerobic cultivations, investigation of gas transfer limitations and substrate depletion	[76, 77]
Ethylene	Plant hormone ethylene released by parsley cells	[78]
Hydrogen	Hydrogen production by anaerobes	[79]
Methane	Methane consumption by bacteria	[80]

## 5 Pitfalls and Resulting Advice for Future Shake Flask Cultivations

The presentation of the current state of research in Chapters 2 to 4 shows that a great deal of research has been carried out on shake flask cultivations, particularly in the last 25 years. However, this knowledge is often not applied or only applied inadequately, which is why some pitfalls during early process development in shake flasks and the conclusions we draw from these for future experiments are discussed below.

### 5.1 Choice of Shake Flask Components

In the first step and before the setup of a cultivation, suitable components for the process should be chosen. In terms of shake flask cultivations, mainly two parts can be changed: the flask and the sterile barrier.

#### 5.1.1 Selection of Sterile Barrier

In contrast to cultivations in a stirred tank reactor, the supply with gas, in particular oxygen, in conventional shake flasks is not achieved by active gassing with bubbles. Instead, aeration takes place at the phase border between the headspace of the flask and the liquid, while the oxygen supply of the headspace from the environment takes place through the sterile barrier in the neck of the flask. A sufficient transfer of oxygen through the sterile barrier must therefore be ensured to prevent this from being the limiting step of gas transfer [14]. Insufficient oxygen supply has been observed for Parafilm and aluminum foil, as they facilitate only little to no gas exchange [17, 23]. Consequently, their use should be avoided to prevent oxygen limitations, but is nevertheless prevalent [81]. The resulting oxygen limitation is not occurring in other scales, leading to unreliable results for scale‐up.

An opposite effect can occur if the airflow into the flask is no longer based on purely diffusive transport but on a convective flow. This effect was described for aluminum caps by Mrotzek et al. [23]. Due to the heightened risk of contamination from convective flow into the flask, we advise against using aluminum caps and rather recommend the use of cotton plugs, which are known for their sterility as well as their sufficient and well‐characterized gas transfer.

#### 5.1.2 Use of Baffled and Unbaffled Shake Flasks

In terms of choosing a shake flask, two different kinds can be chosen: baffled flasks and unbaffled flasks. Shortly after the first use of shake flasks for the cultivation of microorganisms, the design was modified to improve process performance [82]. The use of baffles was intended to improve the power input, transfer rates, and mixing. For that purpose, baffled flasks are still used today [24, 83]. However, their use might result in negative side effects like wetting of the sterile plug, possibly leading to contamination. The reduced reproducibility of the experiments is connected to the varying design of the baffles depending on the manufacturer [24]. The number, size, and geometry of the baffles in combination with the cultivation conditions determine their overall effect [84, 85]. Because of the inconsistent design of baffles, the flow regime of the medium is influenced irreproducibly. Standard calculations and even adapted calculations for baffled shake flasks are difficult to apply, so complex computational fluid dynamic models might be necessary for a reliable simulation of the process [14]. Another negative side effect is the potential formation of out‐of‐phase conditions, see Chapter 3.2. In those cases, the power input is too low to accelerate the liquid against the added baffle resistance [41, 86]. Under the right conditions, a significant improvement of the process can be observed, but in most cases, we conclude that the use of baffled shake flasks should be avoided because of the presented challenges.

### 5.2 Relevance of Process Parameters on Introducued Phenomena during the Process

The different process parameters of shake flask cultivations that were introduced in Chapter 2.2 have a huge impact on the performance of a process and should be kept in mind during early process development. In many publications, process parameters are not documented completely, and it seems like they are not optimized for the conducted process, randomly chosen, or set according to a standard of the working group.

#### 5.2.1 Relevance of Evaluating the Oxygen Supply

**FIGURE 3:**
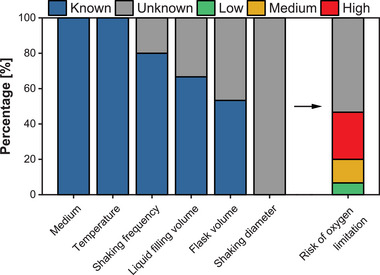
Cultivation parameters provided in publications on shake flask fermentations with *Escherichia coli* and thereof calculated risk of oxygen limitation. Scopus database searched for “*scherichia coli*” and “production” in “Chemical engineering”. The search was made on July 1, 2024, using https://www.scopus.com/. Only research conducted in non‐baffled shake flasks and with the goal of aerobic production was considered for evaluation. Cultivations for genetic engineering purposes were not considered. The most recent 15 articles matching all criteria were analyzed. The OTR_max_ was calculated using Equation (21) [25]. If not explicitly provided in the publication, a shaking frequency of 250 rpm and a shaking diameter of 25 mm were assumed for calculation. In cases where information on the shake flask dimensions or liquid filling volume was missing, the OTR_max_ could not be calculated. The risk of oxygen limitation was categorized as low (>20 mmol∙L^−1^∙h^−1^), medium (15–20 mmol∙L^−1^∙h^−1^), and high (<15 mmol∙L^−1^∙h^−1^) based on the OTR_max_ [26, 87].

The importance of a sufficient oxygen supply for aerobic cultivations is well‐established and extensively documented in the scientific literature. Despite this understanding, many publications in the field of biotechnology lack detailed information on the cultivation conditions necessary to ensure adequate oxygen supply or employ inadequate cultivation conditions that could potentially lead to oxygen limitation. A review of recent publications reveals that medium composition and cultivation temperature are consistently reported, indicating that most researchers prioritize these factors. However, other essential process parameters influencing oxygen transfer, such as shaking frequency, flask dimensions, and liquid filling volume, are frequently omitted, see Figure 3. These omissions hinder the reliable replication of the experimental setup by peers. The analysis also revealed that only a minority of publications provided suitable cultivation conditions to ensure a sufficient oxygen supply.

To ensure sufficient oxygen supply and assess the risk of oxygen limitation, the OTR_max_ should be calculated for the specified process parameters and compared with the actual expected OTR of the process. For this calculation, Equation (21) can be used, or alternatively, the OTR calculator provided by Kuhner (https://kuhner.com/de/otr‐calculator/), which offers a user‐friendly, visual implementation of Equation (21). Only parameters that are already known—osmolality, shaking frequency, liquid filling volume, shaking diameter, maximum inner flask diameter, pressure, and oxygen mole fraction in the gas phase—are required for the calculation. Although all parameters in Equation (21) should be selected sensibly before setting up a cultivation, the following are most accessible for adjustment and most recommended: the shaking frequency (increase to enhance oxygen supply), the liquid filling volume (decrease to enhance oxygen supply), and the shaking diameter (increase to enhance oxygen supply). The improved oxygen supply of all three parameters is due to the increased ratio between the surface area of the liquid to the gas phase and the volume of the liquid phase.

#### 5.2.2 Relevance of Evaluating the Power Input

Next to the volumetric mass transfer coefficient as a measure of oxygen supply, the power input is the most commonly used scale‐up parameter for biotechnological processes [88]. Therefore, a well‐founded characterization in early process development is important. For a long time, it was assumed that the power input in shake flasks was very low compared to stirred systems. However, Büchs et al. showed that this assumption cannot be made in general [30, 31].

With regard to the average volumetric power input, comparable values can be achieved for the shake flask when the corresponding process parameters in the stirred tank reactor are adjusted accordingly. Consequently, a characterization of the process in terms of the average volumetric power input is also possible in early process development and is strongly recommended. According to Equation (1), the average volumetric power input is influenced by the modified Newton number for shake flasks, the liquid density, the shaking frequency, the maximum inner flask diameter, and the liquid filling volume. Typically, only the last three are modified for a targeted adjustment of the power input, as they are more accessible for modification: An increased shaking frequency, a larger inner flask diameter, and a lower liquid filling volume lead to a higher average volumetric power input. The increase in the average volumetric power input is driven by the changed ratio between the liquid filling volume and the liquid's friction area in contact with the flask wall, which primarily determines the power input in a shake flask.

One should always be aware that varying these parameters for other purposes, for example, to change the oxygen supply, also influences the power input.

For the maximum power input, on the other hand, larger differences between the scales can be observed. According to Equation (5), under laminar flow conditions, which are present in most shake flask cultivations, the average volumetric and maximum power input are equal. Based on this and stated by Peter et al., the maximum power input is only influenced by the shaking frequency and the maximum inner flask diameter [33]. An increase in either parameter leads to a higher maximum power input in the shake flask. In the stirred tank reactor, however, the maximum power input is significantly larger, which can have a strong influence on the process. Since the maximum power input or the maximum energy dissipation rate is a measure of the hydromechanical stress, it is a crucial influence factor for processes with sensitive cells like filamentous growing organisms or mammalian cells. Their performance in a production process is strongly dependent on cell viability and morphology making a suitable adjustment of the maximum power input crucial. The difference in maximum power input between scales can lead to deviating results in the shake flask and stirred tank reactor. Based on these findings, we strongly advise to always adequately assess the power input, including both the average and the maximum power input, along with the associated effects.

#### 5.2.3 Relevance of Evaluating the In‐Phase/Out‐of‐Phase Phenomenon

As Büchs et al. already described in 2001, the in‐phase/out‐of‐phase phenomenon is a “hitherto unknown phenomenon” [41], which is true until today since only 0.24 % of all shake flask publications contain this term (Google Scholar searched for “shake flask” compared to “shake flask” and “out‐of‐phase”. The search was made on August 2, 2024, using https://scholar.google.com/). Inadequate selection of process parameters can result in the liquid being out‐of‐phase and thereby strongly influence processes in various ways. Cultivations that behave out‐of‐phase have various negative implications: oxygen limitation, insufficient mixing, and a reduced power input [30, 41]. These consequences strongly affect the growth and performance of the process, so consideration of the phase conditions in early process development is essential and highly recommended. Otherwise, it might lead to the selection of suboptimal strains, which perform better under these limiting conditions than the actual best‐performing strains. The scaled‐up production process will most likely be conducted in stirred tank reactors without these limitations, possibly resulting in a process performance deviating from those observed shake flask scale [89].

To assess the risk for the out‐of‐phase phenomenon, the calculation of the Phase number, see Equation (10), before the cultivation is recommended. As described in Chapter 3.2, Ph > 1.26 and Fr_a_ > 0.4 prevent out‐of‐phase behavior. Impacting process parameters are considered in the equation and should be adapted as follows if there is a risk for the out‐of‐phase phenomenon: increased shaking frequency, increased liquid filling volume, and increased shaking diameter. Another important factor, especially due to its variability throughout the experiment, is the viscosity of the liquid [46, 90]. The initial viscosity, the amount and morphology of the cells, as well as a possible increase in viscosity due to product formation, might lead to a change in viscosity over time. Especially cultivations with filamentous organisms or cultivations‐producing polymers are known for their increasing viscosity over time. This can result in an out‐of‐phase phenomenon even with favorable initial cultivation conditions and should be avoided to guarantee optimal mixing and gas transfer. To prevent the increase in viscosity from impacting the performance, a lower Phase number of 0.91 should be applied for cultivations with high viscosity conditions by setting the process parameters, such as the shaking frequency, appropriately [90, 91].

### 5.3 Insights from the Application of Online Monitoring Devices

Once suitable components have been selected and the appropriate process parameters have been set, the process can be conducted. Here, different online monitoring devices can enable a highly time‐resolved tracking of the process which could not be ensured by offline sampling. Observing the most important process parameters in small‐scale is crucial for a successful scale‐up. Otherwise, changes in these parameters between the scales could impair the process after scale‐up and extensive screening efforts could yield suboptimal processes in the next scale. Consequently, we highly recommend online monitoring of the most critical process parameters in small‐scale, although it might demand considerable effort and come along with higher investment costs. Since all presented online monitoring techniques are non‐invasive, their integration has no direct influence on the process that would oppose their application. Obviously, a critical evaluation of the derived data is always necessary. The following final section presents typical examples in which online monitoring can be particularly beneficial for a process and thus underpins our recommendation to use them.

#### 5.3.1 Insights from Online Monitoring of the Oxygen Transfer

Online monitoring of the OTR is a powerful tool for detecting and mitigating unwanted oxygen limitations, as demonstrated by Losen et al. and Silberbach et al. [92, 93]. However, the information provided by the OTR offers various further insights.

Sufficient amounts of essential nutrients are vital for efficient microbial growth. OTR measurements reveal significant deviations in cultures lacking key nutrients, such as nitrogen or phosphate, compared to conditions of unlimited growth [94, 95, 96]. Lack of nutrients can therefore be easily detected in the OTR data and addressed via targeted supplementation experiments [95]. Moreover, online measurement of the OTR is essential for evaluating complex raw materials, such as yeast extract, and ensuring production consistency. Variations in yeast extracts from different suppliers or batches necessitate the supplementation of amino acids or other micronutrients to achieve consistent and comparable results [6, 97]. Another major uncertainty in the use of complex substrates is the bioavailability of contained nutrients. A simple example would be the provision of unsuited sugar polymers, such as cellulose, to strains lacking cellulolytic enzymes [98]. More complex problems arise when secondary substrates, such as nitrogen or phosphate are affected [96]. Online monitoring of the OTR allows for quantitative evaluation of the amount of bioavailable nutrients by correlating oxygen consumption and available substrate [9, 96, 99].

During screening and process development, product titers are often measured once at the end of the cultivation. However, many organisms will start to consume the desired product when the initial carbon source, for example, glucose, is depleted: Examples include degradation of malic acid by *Ustilago* species, intracellular oil by oleaginous microorganisms, and lysine by *Corynebacterium glutamicum*, or decrease in enzyme content and activity in *Escherichia coli* [67, 68, 100, 101]. Online monitoring of the OTR allows for sampling directly after sugar depletion when the product titer is at its maximum. Wrong decisions in strain selection can hereby be avoided.

#### 5.3.2 Insights from Online Monitoring of the pH Value

In the first step, the correct determination of the initial value of the pH value is essential [102]. In most shake flask cultivations, the pH value is typically not regulated during cultivation. Instead, it is set at the start and buffered to maintain relative stability within a range favorable to the organism. However, monitoring whether the pH value remains within this desired range throughout the process is often overlooked, despite its importance. This contrasts significantly with stirred reactors, where the pH value is continuously and strictly controlled. In contrast to the buffering approach, Scheidle et al. regulated the pH value in shake flasks using a polymer‐based release system that gradually releases pH‐influencing compounds [103]. Online monitoring of the pH value as an application for production processes is rare in literature. Ganjave et al. measured the pH value during high cell density cultivations of *Escherichia coli* in shake flasks and could observe changes over time but did not discuss it further [104]. It can be essential to monitor the pH value for identification of the present metabolism, such as the formation of by‐products like acids. Strategies have now been developed that enable pH value regulation [95]. Coupled with online monitoring, this potentially allows for regulation similar to the one in stirred reactors.

#### 5.3.3 Insights from Online Monitoring of the Biomass

Biomass growth is essential for almost all biotechnological processes, which is why online monitoring of the biomass content can give crucial insights into the state of a cultivation. Numerous studies have utilized online biomass monitoring to evaluate growth rates for comparing production processes. For instance, Wefelmeier et al. tracked biomass during *Ogataea polymorpha* cultivations to optimize poly‐lactic acid production on methanol [105]. Similarly, Bruder et al. assessed the growth rates of various *Saccharomyces cerevisiae* strains with industrial relevance to compare their performance [106]. However, some effects can interfere with the biomass measurement in shake flasks that were described in Chapter 4.1.4: The monitoring is based on an optical measurement method. As a result, the presence of, for example, insoluble substrates and products or the formation of foam can influence the measurement and result in inaccurate and unreliable values [107, 108]. For filamentous organisms, the optical measurement does not always correlate only with the quantity of the biomass, but also with the morphology of the cells. This should be taken into account when assessing the results [108]. On the other hand, this influence on the measurement could also be used to draw conclusions about morphological changes or foam formation.

## 6 Conclusion

In this review, we summarized various research efforts that have been made regarding cultivations in shake flasks in recent years. In the first step, the various process parameters of shake flask cultivations and their impact on different physical effects and phenomena in the shake flask were presented. It was shown that shake flasks have been fully characterized in terms of their power input since the beginning of the millennium. The similarities and differences between the volumetric and maximum power input compared to other scales were highlighted. Essentially, it was found that the maximum power input, in particular, exhibits significant quantitative differences compared to stirred tank reactors. As initially postulated, the in‐phase/out‐of‐phase phenomenon is largely unknown, which is reflected in the number of published publications showing the same trend. The fundamentals of this phenomenon, its effects, and its relevance primarily in the cultivation of viscosity‐altering processes were described. Temperature and mass transfer were also considered, with a focus on the often limited gas transfer from the gas phase to the liquid phase. With regard to the transfer of oxygen, which is crucial for nearly all biotechnological processes, it was found that cultivations in shake flasks are frequently carried out under oxygen‐limited conditions. By selecting suitable process parameters, such as shaking frequency and liquid filling volume, this issue can be avoided in most cases if desired. It is important to pay close attention to this for reliable and scalable results. Furthermore, the choice of the components of the shake flask was considered. To ensure adequate oxygen supply for cultivation and to prevent contamination, it is advisable to avoid using sterile barriers such as aluminum foil, Parafilm, and aluminum caps. Additionally, the disadvantages of baffled flasks, which are often used to improve oxygen supply, were discussed: Their inconsistent design leads to insufficiently reproducible results and carries the risk of the out‐of‐phase phenomenon. Both, the gas transfer in the flasks and its impact on the selection of the sterile barrier, were examined. Finally, several examples illustrating the advantages of online monitoring in shake flasks were presented. It was generally found that there are various advantages associated with the use of online monitoring devices, allowing users to benefit from the high density of data.

## Conflicts of Interest

The authors have declared no conflicts of interest.

## Data Availability

Data sharing is not applicable to this article, as no new data were created or analyzed in this study.
